# Dissecting hypertonicity‐ and NFAT5‐dependent gene expression programs in mpkCCD cells

**DOI:** 10.14814/phy2.70976

**Published:** 2026-06-19

**Authors:** Kristina Engel, Dmitry Chernyakov, Moritz Pernecker, Shobika Karuppusamy, Timm Schreiber, Bayram Edemir

**Affiliations:** ^1^ Department of Medicine, Hematology and Oncology Martin Luther University Halle‐Wittenberg Halle (Saale) Germany; ^2^ Department of Physiology and Pathophysiology Center for Biomedical Education and Research (ZBAF), Witten/Herdecke University Witten Germany

**Keywords:** hypertonicity, mpkCCD, NFAT5

## Abstract

The corticomedullary osmotic gradient between renal cortex and medulla induces a specific spatial gene expression pattern. The factors controlling these differences have not been fully addressed. A hypertonic environment leads to the activation of nuclear factor of activated T‐cells 5 (NFAT5), which regulates the expression of osmoprotective genes. While NFAT5 function under hypertonic conditions has been extensively studied, its contribution to basal gene regulation remains unclear. We used murine principal kidney cortical collecting duct (mpkCCD) cells, induced functional deletion of NFAT5, and performed gene expression profiling to identify genes that are differentially expressed under isotonic and hypertonic cell culture conditions. Hypertonic stress induced extensive transcriptional changes in control cells, which were markedly altered in NFAT5‐deficient cells. Furthermore, a comparison of the mpkCCD transcriptomes with gene expression profiles from the renal cortex and inner medulla of control and principal cell‐specific NFAT5 knockout mice revealed a partial overlap in hypertonicity‐associated and NFAT5‐dependent gene expression patterns. In both conditions, the expression of known NFAT5 target genes, like *Aqp2* and *Ranbp3l,* was downregulated. These findings support the use of mpkCCD cells as a complementary model for studying NFAT5‐associated gene regulation under controlled in vitro conditions.

## 1 INTRODUCTION

Urine‐concentration by the mammalian kidney requires the generation of an interstitial osmotic gradient to provide the driving force for water absorption from the renal collecting ducts (CD) (Sands & Layton, 2009). This osmotic gradient is generated by the active transepithelial reabsorption of NaCl (Dantzler et al., 2014; Fenton & Knepper, 2007; Pannabecker et al., 2004; Sands & Layton, 2014). Urine osmolality and the osmolality of the inner medulla (IM) interstitium can reach up to 1200 mosmol/kg in humans and around 4000 mosmol/kg in mice (Fenton & Knepper, 2007; Sands & Layton, 2014). The cells in the IM are challenged by a hypertonic environment due to the high basolateral NaCl and urea concentrations. However, for the majority of cells, hypertonic stress can cause DNA damage and induce cell death (Burg et al., 2007), but the cells have developed mechanisms to adapt and maintain their function. For example, cells accumulate compatible organic osmolytes such as taurine, myo‐inositol, betaine, and sorbitol. This accumulation is mediated by the actions of the myo‐inositol transporter (SMIT or *SLC5A3*) (Miyakawa et al., 1999), the sodium coupled betaine transporter (BGT1 or *SLC6A12*) (Ito et al., 2004; Miyakawa et al., 1998), the sodium coupled taurine transporter (TauT or *SLC6A12*) (Ito et al., 2004) or via enzymes such as aldose reductase (AR) (Grunewald et al., 1998). Hypertonicity also induces the expression of heat shock protein 70 (*HSP70*) (Aramburu et al., 2006; Burg et al., 2007; Jeon et al., 2006), which protects cells from undergoing apoptosis (Woo et al., 2002).

The central regulator for most of these hypertonicity‐induced processes is the nuclear factor of activated T cells 5 (NFAT5, also known as tonicity‐responsive enhancer‐binding factor TonEBP). This transcription factor is activated under hypertonic conditions, translocates into the nucleus, and induces the expression of genes that are involved in the accumulation of organic osmolytes, the expression of *HSP70*, different urea transporters, and the water channel aquaporin‐2 (AQP2) (Fenton et al., 2002; Hasler et al., 2006; Nakayama et al., 2000).

In contrast, the unique hypertonic environment controls the expression levels of cell‐ and segment‐specific genes. In primary cultivated inner medullary collecting duct (IMCD) cells, the hypertonicity of the cell culture medium induced the expression of genes like aquaporin‐2 (*Aqp2*), the ran binding protein 3 like *(Ranbp3l)*, and many others (Schulze Blasum et al., 2016). Gene expression analysis has demonstrated that genes induced by hypertonicity in cultured IMCD cells are preferentially expressed in nephron segments that are physiologically exposed to a hypertonic interstitium (Chen et al., 2021; Lee et al., 2015), indicating a link between extracellular osmolality and segment‐specific gene expression. Analysis of single cell gene expression (scSeq) from mouse kidneys showed that the expression levels of genes such as *Aqp2* or *Ranbp3l* are highest in cells that are localized in the inner medullary segments (Ransick et al., 2019). These studies show that the hypertonic environment is not only the driving force for generating a concentrated urine but also controls the segment specific gene expression.

Although NFAT5 actions are known to be induced by hypertonicity in the kidney, it is unclear whether it plays a role independently of hypertonic challenge and a systematic analysis of NFAT5 mediated gene expression in the kidney or renal cell lines is missing. One possible explanation for this is that global NFAT5‐deficient mice show high mortality due to impaired kidney and heart development (López‐Rodríguez et al., 2004; Mak et al., 2011).

In this study, we aimed to test the hypothesis that not only the cellular origin but also the environmental hypertonicity can modulate spatial gene expression in the kidney and test the impact of NFAT5 on this gene expression pattern.

We used RNA‐seq to assess gene expression profiles in mpkCCD cells with CRISPR/Cas9 mediated deletion of NFAT5 in the same cells (*Nfat5*‐KO). We used this cell line because it originates from the principal cells of the collecting duct and we have principal cells in the renal cortex (isotonic) and renal inner medulla (hypertonic). The results show that hypertonic cultivation of cells originated from renal cortex induces a gene expression profile that correlates with cells that are localized in the inner medulla. And this study represents the first analysis of NFAT5 mediated gene expression analysis in mpkCCD cells and shows the importance of NFAT5 under both hypertonic and isotonic cell culture conditions.

## 2 RESULTS

### 2.1 Loss of NFAT5 affects gene expression under isotonic cell culture conditions

**FIGURE 1:**
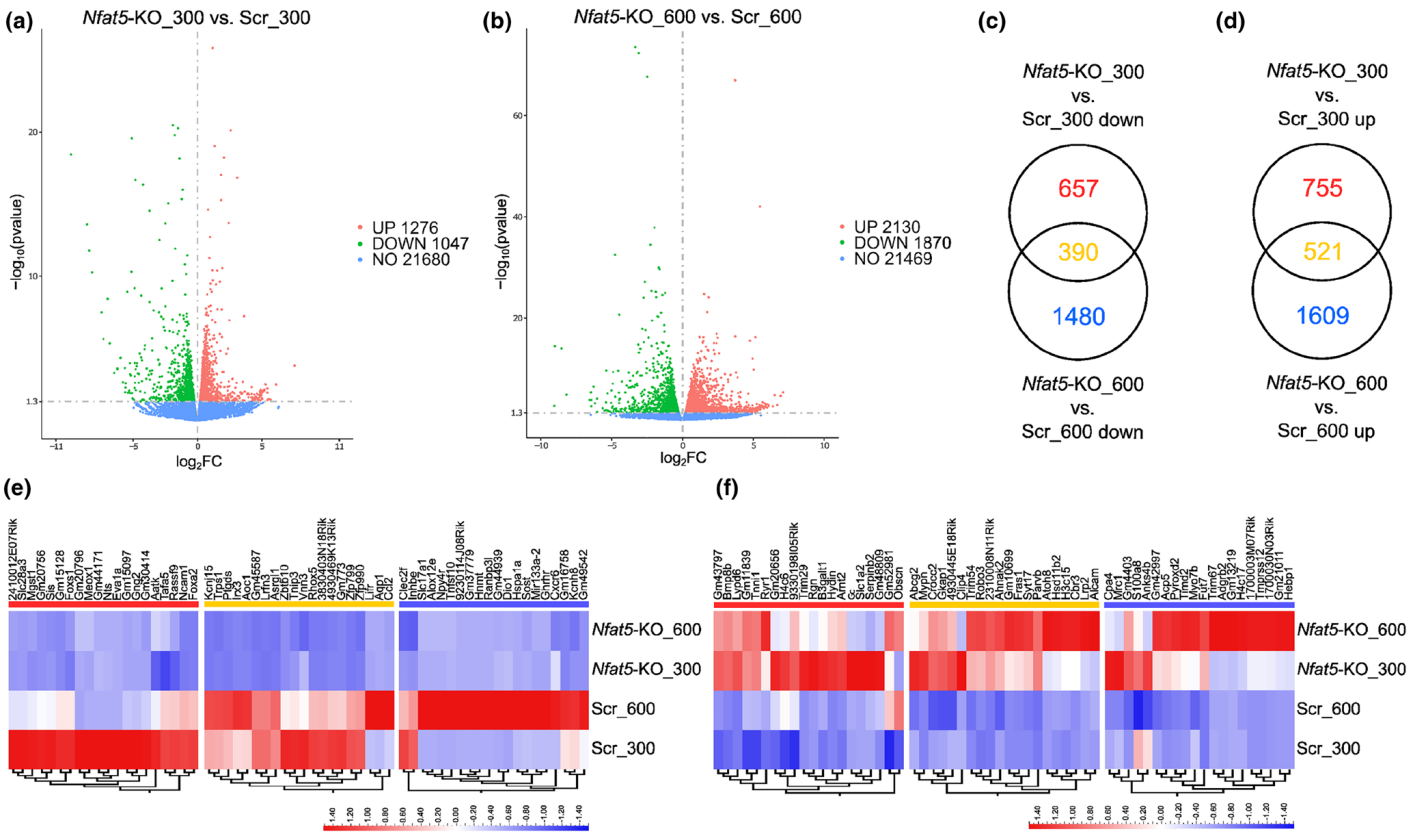
Differentially expressed genes in *Nfat5*‐KO cells. The Volcano plots show the number of differentially expressed genes (DEGs) in *Nfat5*‐KO cells compared to scramble (Scr) cells under isotonic (a, 300) and hypertonic (b, 600) cell culture conditions. The Venn diagram shows the number of common and uniquely downregulated genes (c) or upregulated genes (d) in *Nfat5*‐KO cells under isotonic and hypertonic cell culture conditions. The top 20 downregulated genes from each intersection were used for hierarchical clustering to visualize differences in gene expression levels as *z*‐scores (e). The same analysis was performed for upregulated genes (f).

To assess NFAT5‐dependent gene regulation under basal conditions, we compared the gene expression profiles of *Nfat5*‐KO and Scr cells under isotonic culture conditions. We used CRISPR/Cas9 induced NFAT5 knockout (*Nfat5*‐KO) and scramble guide RNA generated control mpkCCD cells (Scr) as described previously (Chernyakov et al., 2021). *Nfat5*‐KO and Scr cells were cultivated either at 300 mosmol/kg (isotonic) or 600 mosmol/kg (hypertonic) cell culture conditions for 5 days. Total RNA was isolated and processed for RNA‐seq. Principal component analysis and Pearson correlation (Figure S1a,b) confirmed high consistency between biological replicates and clear clustering by genotype and tonicity, indicating robust data quality. We performed comparative gene expression profiling to identify differentially expressed genes (DEG) that were affected by the deletion of NFAT5 or by both osmotic conditions. In *Nfat5*‐KO cells, 1276 DEGs were upregulated and 1047 DEGs were downregulated compared to Scr cells under isotonic cell culture conditions (Figure 1a). The numbers of DEGs were 2130 and 1870, respectively, when the cells were cultivated under hypertonic conditions (Figure 1b). The complete lists of DEGs are provided as Files S1 and S2. Intersection analysis showed that 521 DEGs were commonly upregulated and 390 DEGs were commonly downregulated in *Nfat5*‐KO cells, independent of environmental tonicity (Figure 1c,d). These genes may represent NFAT5 target genes that are generally up‐ or downregulated by NFAT5 activity. The comparison also showed, that the expression of several genes was uniquely affected by the loss of NFAT5 and depended on environmental tonicity. For example, 1609 DEGs were upregulated and 1480 DEGs were downregulated in *Nfat5*‐KO cells under hypertonic cell culture conditions (Figure 1c,d). The complete list of genes from this comparison is provided as File S3. As described above, we used the top 20 DEGs from all intersections and heatmaps after hierarchical clustering to visualize the expression levels in Scr and *Nfat5*‐KO cells (Figure 1e,f).

**FIGURE 2:**
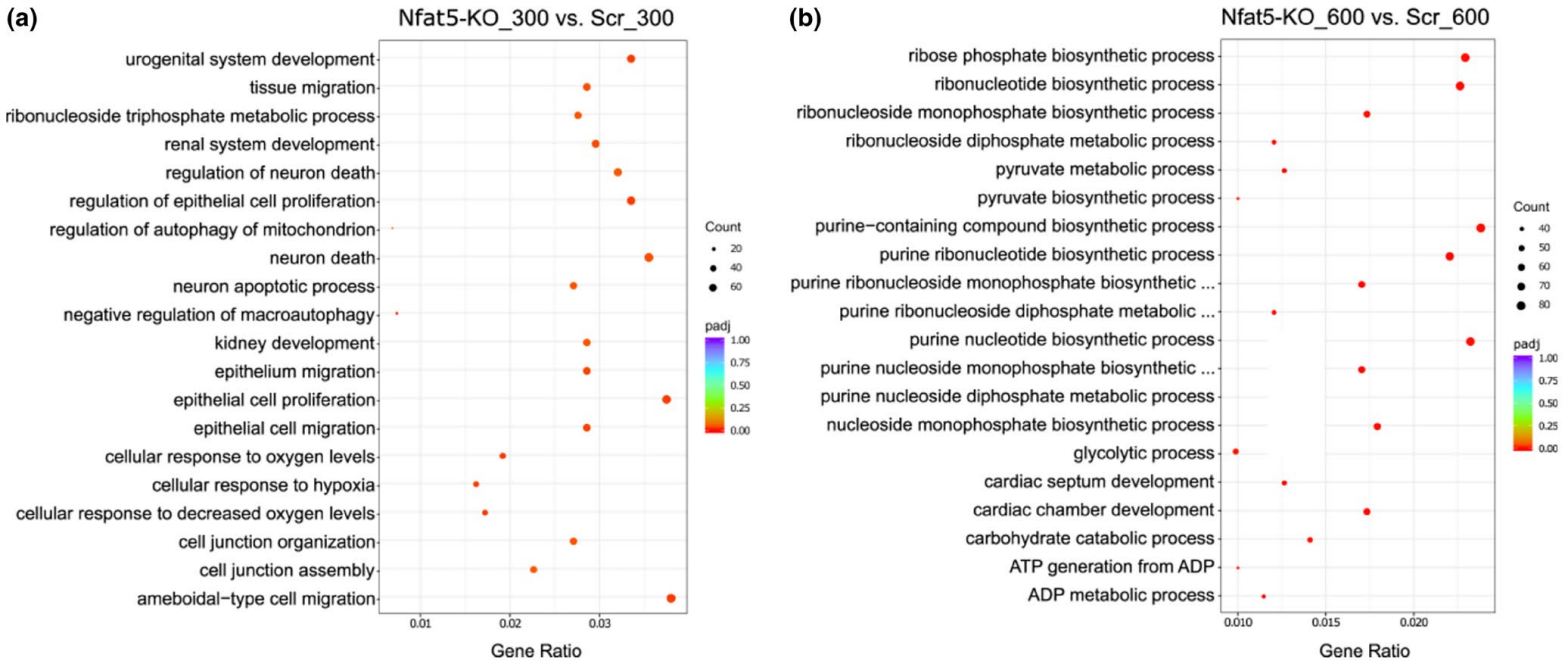
Enriched pathways in *Nfat5*‐KO cells. Under isotonic conditions (a) shows enriched biological pathways and gene ontology (GO) terms associated with differentially expressed genes after loss of NFAT5, while (b) displays enriched pathways and GO terms linked to differentially expressed genes associated with loss of NFAT5 under hypertonic conditions.

Under isotonic conditions, loss of NFAT5 led to enrichment of pathways related to migration, proliferation, and development processes (Figure 2a). In contrast, under hypertonic conditions, NFAT5 deficiency altered ribonucleotide and metabolic processes (Figure 2b).

The results demonstrate that loss of NFAT5 function is associated with massive transcriptional changes already under isotonic cell culture conditions, indicating a role for NFAT5 in basal gene regulation. We identified genes that are differentially expressed by loss of NFAT5 function, independent of environmental tonicity, and genes that were either up‐ or downregulated uniquely under isotonic or hypertonic cell culture conditions.

### 2.2 Hypertonicity‐induced gene expression changes in mpkCCD cells and their modulation by NFAT5

**FIGURE 3:**
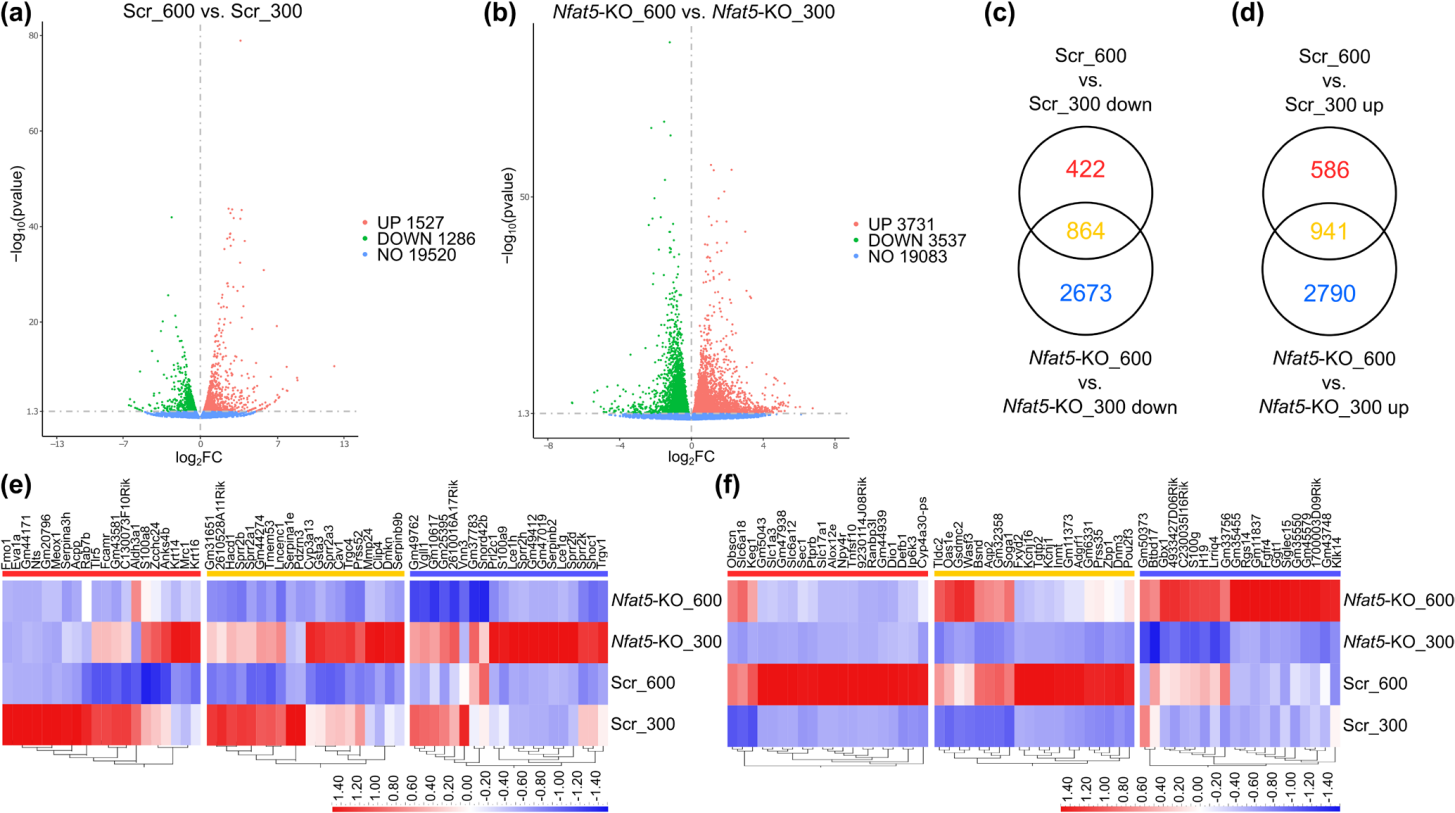
Effect of hypertonicity on gene expression in scramble (Scr) and *Nfat5* knockout (*Nfat5*‐KO) cells. The Volcano plots show the number of DEGs under hypertonic (600) cell culture conditions in Scr (a) and *Nfat5*‐KO cells (b) in comparison to isotonic conditions (300). The Venn diagram shows the number of common and uniquely downregulated (c) or upregulated genes (d) in Scr and *Nfat5*‐KO cells under hypertonic cell culture conditions. The top 20 downregulated genes from each intersection were used for hierarchical clustering to visualize differences in gene expression levels as *z*‐scores (e). The same analysis was performed for upregulated genes (f).

In Scr cells, hypertonicity induced the differential expression of 1527 genes and reduced the expression of 1266 genes (Figure 3a). Interestingly, in *Nfat5*‐KO cells, the number of upregulated DEGs was 3731 and 3537 DEGs were downregulated under hypertonic cell culture conditions (Figure 3b). The complete lists of DEGs are provided as Files S4 and S5. We performed an intersection analysis using the lists of DEGs from Figure 3a,b, and separated for up‐ and downregulated DEGs. Figure 3d shows that 941 DEGs were commonly upregulated in Scr and *Nfat5*‐KO cells by hypertonicity. These genes may be upregulated by hypertonicity, independent of NFAT5 action. However, hypertonicity led to the up regulation of 586 DEGs uniquely in Scr and 2790 DEGs only in *Nfat5*‐KO cells. In the same way we compared the lists of downregulated DEGs. Here we identified 864 DEGs that were commonly downregulated by hypertonicity independent of NFAT5 action (Figure 3c). The complete lists with all common and unique genes are provided as File S6 Among downregulated DEGs, 422 were unique to the Scr and 2673 unique to the *Nfat5*‐KO cells. This indicates, that other factors than NFAT5 are involved in the changes in gene expression under hypertonic cell culture conditions. Furthermore, we used the top 20 DEGs from all intersections to visualize the expression levels using heatmaps after hierarchical clustering. Figure 3e shows the heatmaps for downregulated genes, and Figure 3f for upregulated genes.

**FIGURE 4:**
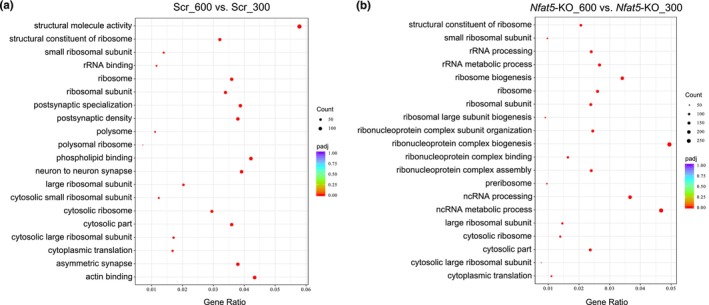
Effect of hypertonicity on enriched pathways in scramble (Scr) and *Nfat5* knockout (*Nfat5*‐KO) cells. Enriched gene ontology (GO) terms are shown related to osmolality‐dependent gene regulation (a), while (b) displays GO terms enriched in differentially expressed genes upon hypertonic stress in NFAT5‐deficient cells.

Functional enrichment analysis revealed that osmolality‐dependent DEGs in Scr cells were mainly involved in structural molecule activity and ribosomal functions (Figure 4a), whereas hypertonic stress in NFAT5‐deficient cells affected pathways related to RNA processing and ribosome‐associated processes (Figure 4b).

**FIGURE 5:**
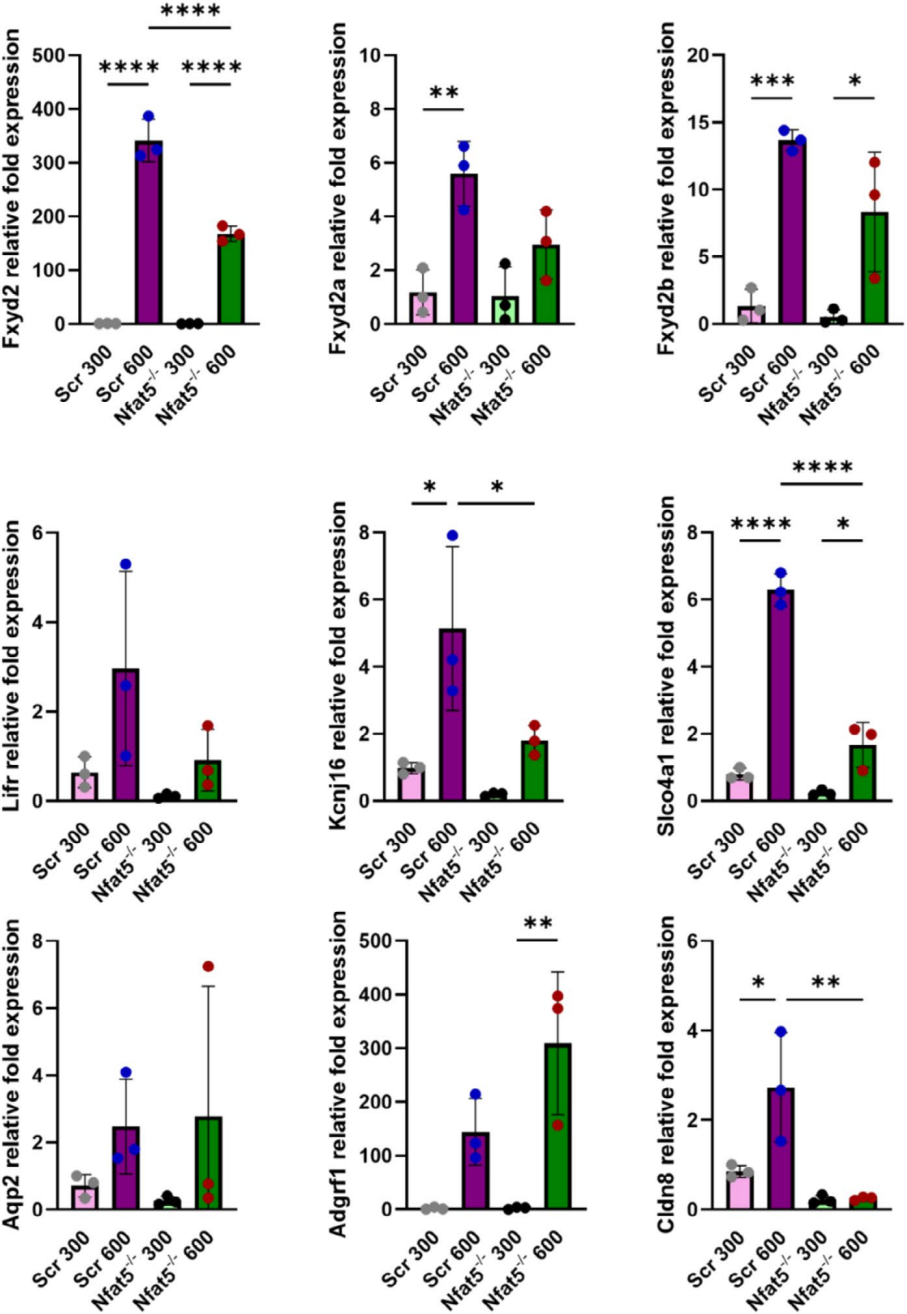
Real time PCR for validation of the NGS Data. Relative expression of *Fxyd2*, *Fxyd2a*, *Fxyd2b*, *Lifr*, *Kcnj16*, *Slco4a1*, *Aqp2*, *Adgrf1*, and *Cldn8* was analyzed by Real Time PCR. Significant differences were analyzed by One‐Way‐Anova (*p < 0.05; **p < 0.01; ***p < 0.001; ****p < 0.0001; *n* = 3).

We performed qPCR validation for selected DEG genes. The results are shown in Figure 5.

### 2.3 Loss of NFAT5 affects inversely the expression of hypertonicity affected genes

**FIGURE 6:**
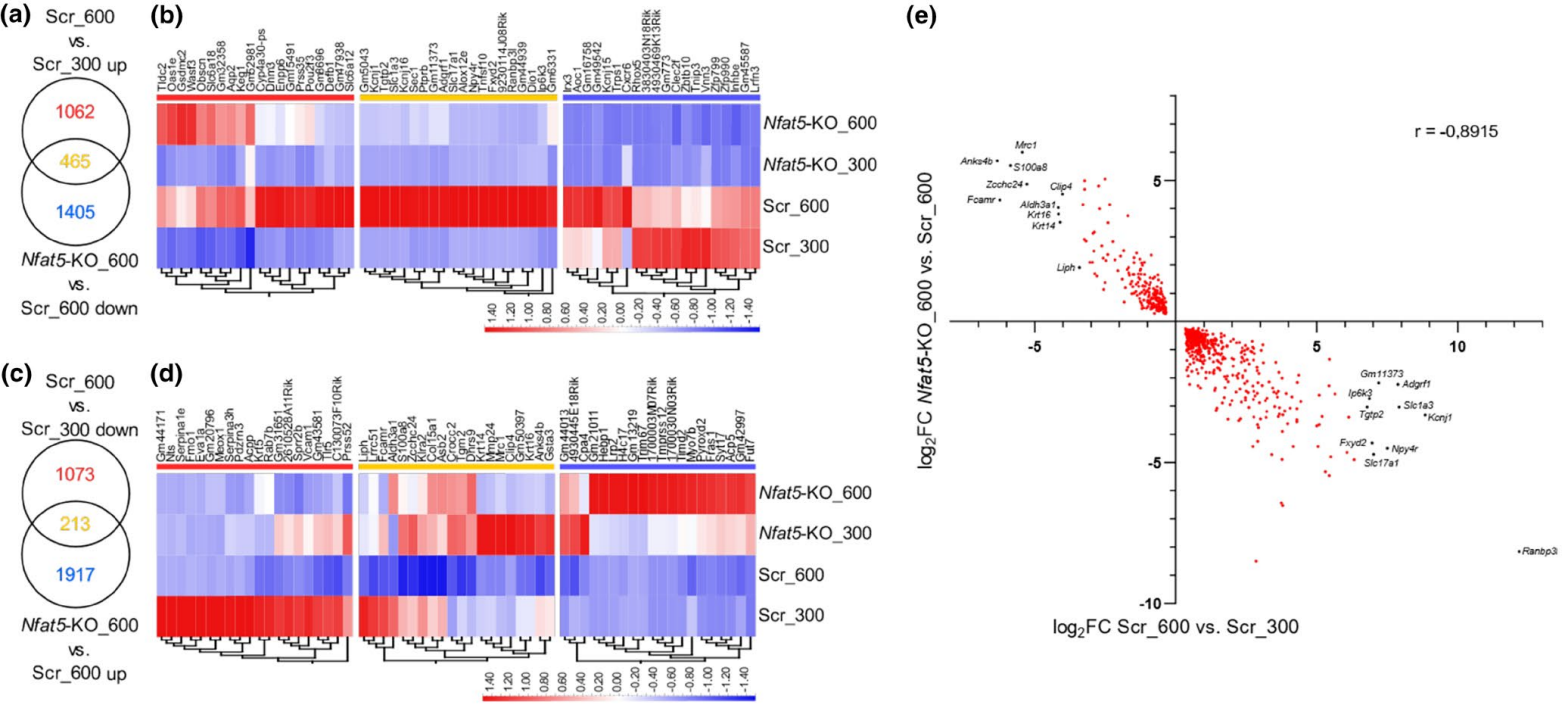
Differentially expressed genes in NFAT5 knockout cells in response to hypertonicity. (a + c) The Venn diagram shows the number of common and uniquely regulated genes under hypertonic (600) conditions and *Nfat5*‐KO effect. In (a), genes are upregulated in Scr cells and downregulated by *Nfat5*‐KO at 600 mosmol/kg. In (c), the opposite regulation is shown with genes downregulated in Scr cells and upregulated after *Nfat5*‐KO. (b) The top 20 differentially expressed genes from each intersection in (a) were used for hierarchical clustering to visualize differences in gene expression levels as *z*‐scores. (d) The same analysis was performed for differentially expressed genes from the intersections shown in (c). Correlation analysis (e) shows genes, which are down‐ or upregulated after hyperosmolarity and regulated in a different direction after *Nfat5*‐KO. Top 10 genes are highlighted.

To further identify and discriminate NFAT5 target genes affected by NFAT5 activity in combination with hypertonicity, we performed an intersection analysis using DEGs in NFAT5‐KO at 600 mosmol/kg, shown in Figure 1b and DEGs after hyperosmolar cultivation in Scr cells shown in Figure 3a. We identified 465 DEGs that are upregulated in Scr cells under hypertonic cell culture conditions and downregulated in *Nfat5*‐KO cells under hypertonic cell culture conditions (Figure 6a). In the same way we identified 213 DEGs that were downregulated under hypertonic cell conditions in Scr cells but upregulated in *Nfat5*‐KO cells (Figure 6c). The complete list is provided as File S7. These data indicate that these genes may represent NFAT5 target genes that are affected by hypertonic cell culture conditions. The heatmap in Figure 6b,d visualizes the expression patterns of the top 20 DEGs from each intersection. Furthermore, the common genes of both Venn analyses (Figure 6a,c) showed a strong anti‐correlation of −0.8915 (Figure 6e). This indicates that the expression of these genes depend on NFAT5 expression.

This comparison showed, that some of the hypertonicity‐induced differences in gene expression levels might be mediated by the action of NFAT5. For example, the expression levels of *Kcnj1*, *Kcnj16* or *Slc17a1* were induced by hypertonicity but downregulated in *Nfat5*‐KO cells. For example, as the expression of *Gsmdc2* or *Slc6a18* is still increased under hypertonic cell culture conditions in *Nfat5*‐KO cells, other factors might be involved in the observed changes in gene expression under hypertonic cell culture conditions.

### 2.4 Differences in gene expression level are also observed in kidneys from *Nfat5*‐KO mice

**FIGURE 7:**
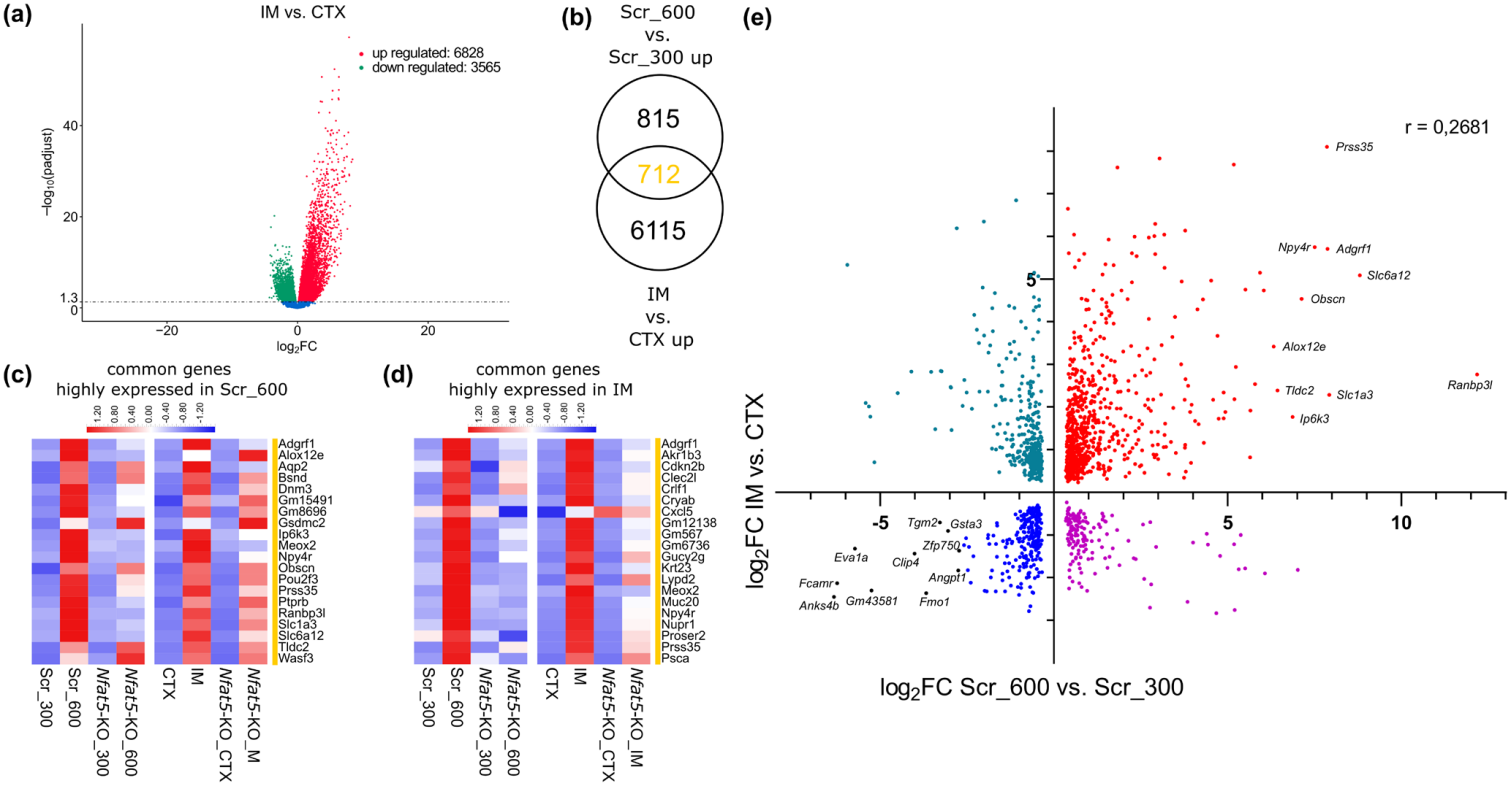
Differentially expressed genes in mpkCCD cells and in the cortex (CTX) and inner medulla (IM) of mice. (a) The volcano plot shows the number of differentially expressed genes (DEGs) in the IM compared to the CTX in mice. (b) The Venn diagram shows the number of common and uniquely upregulated genes in mpkCCD scramble (Scr) cells under hypertonic conditions (Scr_600 versus Scr_300 up) and in IM cells (IM versus CTX) from mice (IM vs. CTX up). The top 20 commonly upregulated genes in IM (c) and in Scr_600 (d) were used for hierarchical clustering to visualize differences in gene expression levels as *z*‐scores in Scr and *Nfat5*‐KO mpkCCD cells under isotonic (300) and hypertonic (600) conditions, as well as in CTX and IM cells of control and *Nfat5*‐KO mice (*Nfat5*‐KO_IM and *Nfat5*‐KO_CTX). Correlation analysis (e) shows genes, which are down‐ or upregulated after hyperosmolarity in Scr cells and down‐ or upregulated in hyperosmotic environment in the kidney.

Because the results observed so far were derived from in vitro experiments, we analyzed, wether this might also be evident in vivo. Since isotonic cell culture medium could reflect the situation in the renal cortex and hypertonic medium osmolality the situation in the renal inner medulla, we used data, derived from gene expression analysis using renal cortex (CTX) versus renal inner medulla (IM) from control (Figure 7a) or principal cell‐specific NFAT5 deficient KO‐CTX and KO‐IM mice described in (Engel et al., 2025) and performed intersection analysis. The lists with the DEGs are provided as File S8. We identified 712 DEGs that were upregulated under hypertonic cell culture conditions and had a higher expression in IM compared to CTX (Figure 7b) in the control mice. These data show that in part, the cultivation of mpkCCD cells under hypertonic cell culture conditions affects the expression of the same genes as observed in the IM. The gene lists from this comparison are provided as File S9. We used top 20 DEGs in IM compared to CTX and top 20 common upregulated DEGs in mpkCCD under hypertonic cell culture conditions and generated heatmaps to visualize differences in gene expression levels (Figure 7c,d). The expression level DEGs that were upregulated under hypertonic cell culture conditions (Scr_600) showed reduced expression levels in *Nfat5*‐KO (*Nfat5*‐KO_600) cells. The expression level of these genes were higher in the IM group compared to the CTX group. However, similar to the situation in mpkCCD cells, the expression levels of these genes were downregulated in the IM of *Nfat5*‐KO kidneys. These results indicate that hypertonicity and loss of NFAT5 in mpkCCD cells partially mirror transcriptional changes observed in vivo, particularly for established NFAT5 target genes. These include genes like *Aqp2*, *Ranbp3l*, *Slc6a12* and many others. The correlation analysis showed a lower‐positive correlation of 0.2681 (Figure 7e) but there were also genes, that demonstrated a different direction in Scr cells as in the kidney samples.

## 3 DISCUSSION

The renal corticomedullary osmotic gradient is essential for the generation of concentrated urine. Under cell culture conditions, hypertonicity induces the expression of kidney specific transcripts in primary cultivated IMCD cells (Schulze Blasum et al., 2016). In the kidneys, the osmotic gradient allows a spatial expression pattern of genes that are not present in most other cell types (Hinze et al., 2021). The factors that contribute to this gene expression pattern are not well understood. One factor that can influence gene expression is the cellular identity, which can contribute to spatial differences in gene expression patterns. Principal cells of the collecting duct can be found in the renal cortex and in the renal inner medulla. However, there are differences in gene expression patterns due to the spatial localization of cells (Chen et al., 2017; Ransick et al., 2019). Here we show that the cultivation of the cortical principal cell line mpkCCD under hypertonic cell culture conditions can induce a gene expression profile that correlates with that observed in inner medullary collecting duct cells.

NFAT5 is the master transcription factor for hypertonicity‐induced gene expression and loss of NFAT5 function in the principal cells of the collecting duct is associated with a diabetes insipidus‐like phenotype and massive changes in gene expression (Chernyakov et al., 2022; Petrillo et al., 2022). However, besides the loss of NFAT5 function, the observed changes in gene expression might indirectly be associated with systemic changes, which are associated with differences in renal function or other factors that could induce changes in gene expression. In this study, we also showed that the changes in gene expression observed in NFAT5 deficient mice can be partly observed in a cell culture setting.

The mpkCCD cells have been frequently used to analyze the effect of vasopressin‐mediated signaling on AQP2 regulation (Fenton et al., 2008; Hoffert et al., 2008; Yu et al., 2009). They have also been frequently used for system‐level analysis approaches using proteomic and genomic‐based analysis (Hoffert et al., 2009; Jung et al., 2018; Rinschen et al., 2010). The mpkCCD cells have also been used to analyze the effect of hypertonicity on gene expression (Groß et al., 2020). The study presented here is the first study using NFAT5 deficient mpkCCD cells to identify genome wide DEGs that are up‐ or downregulated under hypertonic cell culture conditions due to loss of NFAT5 function. In contrast to the in vivo situation, we can directly link the observed changes to the loss of NFAT5 function or differences in environmental hypertonicity. Hypertonicity led to massive changes in gene expression in Scr and *Nfat5*‐KO cells. Around 586 genes were uniquely up‐ and 422 uniquely downregulated in Scr cells. This indicates that NFAT5 is involved in the expression of these genes. This includes genes like *Slc6a12* and *Ranbp3l*. *Slc6a12* is coding for beta‐GABA transporter 1 (BGT1) and is a classical osmoprotective gene that has been shown to be upregulated under hyperosmotic conditions in MDCK cells and is a NFAT5 target gene (Burg et al., 2007; Lammers et al., 2005; Miyakawa et al., 1999; Neuhofer et al., 2007; Woo et al., 2000). *Ranbp3l* expression is upregulated under hypertonic cell culture conditions in primary cultivated IMCD cells (Schulze Blasum et al., 2016) and mouse embryonic fibroblasts (Izumi et al., 2015). In both cases, loss of NFAT5 is associated with reduced *Ranbp3l* expression (Chernyakov et al., 2021; Izumi et al., 2015). The present data support the results obtained from other studies and indicate that NFAT5 function is required for the observed upregulation of *Slc6a12, Ranbp3l* and the other genes identified to be uniquely induced in Scr cells under hypertonic conditions. Although we identified common genes that were upregulated in Scr and *Nfat5*‐KO cells, the level of upregulation was different. For example, the expression level of *Fxyd2*, *Adgrf1*, *Aqp2* or *Prss35* was lower in *Nfat5*‐KO cells than in Scr cells (Figure 2f). These may represent genes where the expression is partly modulated by NFAT5. This effect was also confirmed by RT‐qPCR (Figure 5). *Fxyd2* is coding for the gamma subunit of the sodium potassium ATPase and it has been shown that its expression can be induced by osmotic stress in rat proximal tubule derived NRK‐52E cells (Wetzel et al., 2004). The expression of *Aqp2* on mRNA level is regulated by cAMP‐responsive‐element‐binding protein (CREB) (Yasui et al., 1997). However, its expression can also be modulated by other factors like GATA2, ELF5 or NFAT5 (Grassmeyer et al., 2017; Hasler et al., 2006; Petrillo et al., 2022; Yu et al., 2014). A recent study showed that even more factors could contribute to *Aqp2* mRNA expression (Murillo‐de‐Ozores et al., 2026).

The expression of *Kcnj1* and *Kcnj16*, coding for potassium inwardly‐rectifying channel, subfamily J, member 1 (ROMK1) and potassium inwardly‐rectifying channel, subfamily J, member 16, respectively, is increased under hypertonic cell culture conditions. For ROMK1, the contribution of hypertonicity and NFAT5 to its expression level has been shown in isolated thick ascending limb (TAL) segments and TAL mice cells (Gallazzini et al., 2006). Loss of KCNJ16 function is associated with hypokalemia, salt wasting, and sensorineural deafness (Schlingmann et al., 2021). So far, the regulation of KCNJ16 expression by hypertonicity or NFAT5 has not been described and the functional consequences are unknown.

It has been shown that the cultivation of primary IMCD cells under hypertonic cell culture conditions induces the expression of kidney specific genes and that the osmotic gradient between renal cortex and inner medulla contributes to the spatial differences in gene expression (Hinze et al., 2021; Schulze Blasum et al., 2016). Using collecting duct principal cell‐specific *Nfat5*‐KO kidneys, we identified the effect of NFAT5 deletion on renal function and gene expression (Chernyakov et al., 2022; Petrillo et al., 2022). By comparing mpkCCD transcriptomes with gene expression profiles from the renal cortex and inner medulla in the present study, we observed a partial overlap (nearly 50%) between hypertonicity‐induced genes in vitro (mpkCCD cells) and genes enriched in the renal IM in vivo. This finding is consistent with previous studies showing that extracellular osmolality contributes to spatial gene expression patterns along the corticomedullary axis (Hinze et al., 2021). Importantly, many overlapping genes exhibited reduced expression in principal cell‐specific NFAT5 knockout kidneys, supporting the idea that at least part of the corticomedullary gene expression gradient is directly mediated by NFAT5. Nevertheless, the incomplete overlap also highlights the contribution of additional factors present in vivo, including cellular heterogeneity, interstitial signaling, and systemic regulatory mechanisms.

Reduced expression was observed for example for *Meox2*, *Akr1b3* or *Cryab*. For *Meox2*, no specific renal function has been described. *Akr1b3* is coding for aldo‐keto reductase family 1 member B (AR). The action of AR is required for the adaptation of cells to hypertonic stress (Burg et al., 2007; Grunewald et al., 1998; Jeon et al., 2007; Lim et al., 2007). Our data confirmed the results obtained before and shows that the expression of AR is mediated by NFAT5. *Cryab*, coding for crystallin alpha B, is a member of the small heat shock protein (HSP20) (Fujimoto & Nakai, 2010). Also, for *Cryab* no specific renal function has not been described so far. It has been shown that the expression of *Cryab* is induced upon cisplatin treatment and protects against cisplatin nephrotoxicity (Lou et al., 2016). The mentioned study shows, that CRYAB is a stress induced factor and contributes to cellular protection. The increased expression upon hypertonic stress and NFAT5 function could also represent a protective effect. However, if and how CRYAB contributes to cellular protection needs to be shown in further studies. Also, further studies are needed to analyze, if the observed changes in gene expression are also evident on the protein level.

There are also limitations of the present study that we would like to acknowledge. First, the use of clonal CRISPR/Cas9‐generated NFAT5‐deficient mpkCCD cell lines may introduce clonal adaptations that are independent of NFAT5 deletion. Although two independently derived clones showed highly consistent transcriptional profiles and concordant regulation of established NFAT5 target genes, clonal effects could not be completely excluded. Second, the comparison between cultured tubular cells and whole kidney tissue is inherently limited by differences in cellular composition and microenvironment. Our analysis therefore focuses on shared transcriptional trends rather than direct quantitative equivalence. Finally, the present study is based on transcriptomic data, and further work will be required to determine the extent to which the observed changes are reflected at the protein and functional level. Despite these limitations, our data support the use of mpkCCD cells as a controlled in vitro model to dissect NFAT5‐associated transcriptional programs and to distinguish cell‐autonomous effects of NFAT5 from systemic influences present in vivo.

In summary, this is the first study using mpkCCD cells to show the contribution of NFAT5 to hypertonic and non‐hypertonic induced gene expression. We show that the changes in gene expression, either by hypertonicity or loss of NFAT5 function, correlated with the observed changes in gene expression levels in kidneys from control and principal cell specific NFAT5 deficient cells.

## 4 METHODS

### 4.1 Cell culture

The mpkCCD cells were cultivated as described before. We used two CRISPR/Cas9 mediated *Nfat5* deleted mpkCCD (N1 and N3) clones generated before (Chernyakov et al., 2021) and mpkCCD cells that were transduced with a scramble (Scr) non‐targeting gRNA (Chernyakov et al., 2021; Groß et al., 2020). All cells were cultured on standard tissue‐culture plastic at 37°C and 5% CO_2_. The medium osmolality was adjusted to 600 mosmol/kg by the addition of 100 mM NaCl (71376, Sigma Aldrich) and 100 mM urea (U5378, Sigma Aldrich) to the corresponding medium. Cells were exposed to isotonic (300 mosmol/kg) and hypertonic medium (600 mosmol/kg) for 5 days. Life‐cell imaging was used for subjective assessment of cell viability and no sustained loss of viability was observed under these conditions. A combination of sodium chloride (NaCl) and urea was chosen to more adequately mimic the physiological composition of the renal inner medullary interstitium. Both osmolytes contribute to NFAT5 activation.

### 4.2 Mouse strains

To generate mice (strain C57BL/6N) that are deficient in NFAT5 in the principal cell (PC) of the collecting duct (CD), floxed NFAT5 mice (Nfat5^fl/fl^) were crossed with Aqp2‐Cre^+/−^ mice expressing CRE recombinase under the control of the *Aqp2* gene promoter. (Chernyakov et al., 2022; Petrillo et al., 2022). The insertion of the Cre recombinase disrupts one allele of the *Aqp2* gene. Therefore, we used only Aqp2Cre^+/−^Nfat5^fl/fl^ mice with Aqp2‐Cre^+/−^ mice as controls.

### 4.3 RNA isolation and preparation of samples for NGS

The Scr control cells and the two *Nfat5*‐KO cells were cultivated either at 300 mosmol/kg or 600 mosmol/kg (for 5 days). For gene expression analysis using NGS RNA‐sequencing (RNA‐seq), total RNA from mpkCCD cells as well as from renal cortex and renal inner medulla of mouse kidneys was isolated using the Gen Elute Mammalian Total RNA prep kit (RTN350‐1KT, Sigma Aldrich). The quality control, sequencing, and bioinformatics were performed as described (Groß et al., 2020). We used two biological replicates for each condition and each genotype. The data presented here and the raw data are available via Gene Expression Omnibus (Acc. No. GSE326713).

### 4.4 RNA quantification and qualification

RNA degradation and contamination were monitored on 1% agarose gels. RNA purity was checked using the NanoPhotometer® spectrophotometer (IMPLEN, CA, USA). RNA integrity and quantitation were assessed using the RNA Nano 6000 Assay Kit of the Bioanalyzer 2100 system (Agilent Technologies, CA, USA).

### 4.5 Library preparation for transcriptome sequencing

Library preparation and sequencing was performed as described before (Groß et al., 2020). A total amount of 1 μg RNA per sample was used as input material for the RNA sample preparations. Sequencing libraries were generated using NEBNext® UltraTM RNA Library Prep Kit for Illumina® (New England Biolabs, Ipswich, MA, USA) following the manufacturer's recommendations and index codes were added to attribute sequences to each sample. Briefly, mRNA was purified from total RNA using poly‐T oligo‐attached magnetic beads. Fragmentation was carried out using divalent cations under elevated temperature in NEBNext First Strand Synthesis Reaction Buffer (5X). First strand cDNA was synthesized using random hexamer primer and M‐MuLV Reverse Transcriptase (RNase H‐). Second strand cDNA synthesis was subsequently performed using DNA Polymerase I and RNase H. Remaining overhangs were converted into blunt ends via exonuclease/polymerase activities. After adenylation of 3′ ends of DNA fragments, NEBNext Adaptor with hairpin loop structure was ligated to prepare for hybridization. In order to select cDNA fragments of preferentially 150 ~ 200 bp in length, the library fragments were purified with AMPure XP system (Beckman Coulter, Beverly, USA). Then 3 μL USER Enzyme (New England Biolabs, USA) was used with size‐selected, adaptor ligated cDNA at 37°C for 15 min followed by 5 min at 95°C before PCR. Then PCR was performed with Phusion High‐Fidelity DNA polymerase, Universal PCR primers and Index (X) Primer. At last, PCR products were purified (AMPure XP system) and library quality was assessed on the Agilent Bioanalyzer 2100 system.

### 4.6 Clustering and sequencing

The clustering of the index‐coded samples was performed on a cBot Cluster Generation System using PE Cluster Kit cBot‐HS (Illumina, San Diego, CA, USA) according to the manufacturer's instructions. After cluster generation, the library preparations were sequenced on an Illumina platform, and paired‐end reads were generated.

### 4.7 Data analysis and quality control

Raw data (raw reads) of FASTQ format were firstly processed through in‐house scripts. In this step, clean data (clean reads) were obtained by removing reads containing adapter and poly‐N sequences and reads with low quality from raw data. At the same time, Q20, Q30, and GC content of the clean data were calculated. All the downstream analyses were based on the clean data with high quality (Cock et al., 2010; Erlich et al., 2008; Jiang et al., 2011).

### 4.8 Mapping to reference genome

Reference genome and gene model annotation files were downloaded from genome website browser (NCBI/UCSC/Ensembl) directly. Paired‐end clean reads were mapped to the reference genome GRCm38/mm10 using HISAT2 software. HISAT2 uses a large set of small GFM indexes that collectively cover the whole genome. These small indexes (called local indexes), combined with several alignment strategies, enable rapid and accurate alignment of sequencing reads (Altschul et al., 1997; Dobin et al., 2013; Finn et al., 2008).

### 4.9 Quantification

HTSeq was used to count the read numbers mapped of each gene, including known and novel genes. And then RPKM of each gene was calculated based on the length of the gene and reads count mapped to this gene. RPKM, Reads Per Kilobase of exon model per Million mapped reads, considers the effect of sequencing depth and gene length for the reads count at the same time, and is currently the most commonly used method for estimating gene expression levels (Dillies et al., 2013; Trapnell et al., 2010).

### 4.10 Differential expression analysis

Differential expression analysis between two conditions/groups was performed using DESeq2 R package. DESeq2 provides statistical routines for determining differential expression in digital gene expression data using a model based on the negative binomial distribution. The resulting *p* values were adjusted using the Benjamini and Hochberg's approach for controlling the False Discovery Rate (FDR). Genes with an adjusted *p* < 0.05 found by DESeq2 were assigned as differentially expressed (Anders & Huber, 2010). We filtered our results with a Log_2_ fold change (Log_2_FC) ≠ 0 and a *p* < 0.05, independent from a minimum count. Heatmap visualization of statistically differentially expressed genes and z‐score calculations were conducted using Qlucore Omics Explorer 3.9 (Qlucore, New York, NY, USA).

### 4.11 cDNA synthesis and RT‐qPCR

For cDNA synthesis, 1 μg of isolated RNA was used. The expression of the target gene was determined by RT‐qPCR using specific primers, separated by at least one intron on the corresponding DNA as described before (Schulze Blasum et al., 2016). Data acquisition was done with StepOne Software v2.3 and quantified by the 2^−∆∆CT^ method as described (Livak & Schmittgen, 2001). The sequences of the used primers are listed in File S10.

## AUTHOR CONTRIBUTIONS

**Kristina Engel:** Conceptualization; data curation; formal analysis; methodology. **Dmitry Chernyakov:** Data curation; formal analysis; investigation; methodology. **Moritz Pernecker:** Formal analysis; investigation. **Shobika Karuppusamy:** Investigation. **Timm Schreiber:** Formal analysis. **Bayram Edemir:** Conceptualization; data curation; formal analysis; funding acquisition; investigation; methodology; project administration; supervision.

## FUNDING INFORMATION

B.E. was supported by the funds of the German Research Foundation (ED 181/9‐3).

## CONFLICT OF INTEREST STATEMENT

The authors declare no conflict of interest.

## ETHICS STATEMENT

None.

## Supporting information


**File S1.** Nfat5‐KO_300 versus Scr_300 differentially expressed genes (DEG).

**File S2.** Nfat5‐KO_600 versus Scr_600 differentially expressed genes (DEG).

**File S3.** Gene groups corresponding to the Venn diagramm shown in Figure 1C,D.

**File S4.** Scr_600 versus Scr_300 differentially expressed genes (DEG).

**File S5.** Nfat5‐KO_600 versus Nfat5‐KO_300 differentially expressed genes (DEG).

**File S6.** Gene groups corresponding to the Venn diagramm shown in Figure 3C,D.

**File S7.** Gene groups corresponding to the Venn diagramm shown in Figure 6A,C.

**File S8.** IM versus CTX differentially expressed genes (DEG).

**File S9.** Gene groups corresponding to the Venn diagramm shown in Figure 7B.

**File S10.** RT‐qPCR primer sequences.

**Figure S1.** Clustering of RNA‐seq samples based on genotype and tonicity. (A) Principal component analysis (PCA) of RNA‐seq data from mpkCCD cells under isotonic (300 mosmol/kg) and hypertonic (600 mosmol/kg) conditions in scramble control (Scr) and Nfat5 knockout (*Nfat5*‐KO) cells. The three principal components explain 23%, 21% and 13% of the total variance, respectively. Each point represents a single biological replicate. (B) Correlation heatmap based on Pearson correlation coefficients. Samples are primarily clustered by osmotic conditions and genotype, confirming the consistency between biological replicates and the strong transcriptomic differences associated with NFAT5 function and tonicity.

## Data Availability

The raw data of mpkCCD cells presented have been submitted to the public repository GEO number (Gene Expression Omnibus) GSE326713. The raw data of *Nfat5‐*KO and control mice have been submitted under GEO number GSE195881.
