# Supplementary material for: Dissecting hypertonicity‐ and NFAT5‐dependent gene expression programs in mpkCCD cells
Source: Physiol Rep. 2026 Jun 19;14(12):e70976. doi: 10.14814/phy2.70976 (PMC13282263; doi:10.14814/phy2.70976)
Supplement: Supplementary file 11 — Figure S1. Clustering of RNA‐seq samples based on genotype and tonicity. (A) Principal component analysis (PCA) of RNA‐seq data from mpkCCD cells under isotonic (300 mosmol/kg) and hypertonic (600 mosmol/kg) conditions in scramble control (Scr) and Nfat5 knockout (Nfat5‐KO) cells. The three principal components explain 23%, 21% and 13% of the total variance, respectively. Each point represents a single biological replicate. (B) Correlation heatmap based on Pearson correlation coefficients. Samples are primarily clustered by osmotic conditions and genotype, confirming the consistency between biological replicates and the strong transcriptomic differences associated with NFAT5 function and tonicity. [file PHY2-14-e70976-s009.docx]

Loss of nuclear factor of activated T cells 5 (NFAT5) activity on gene expression in mpkCCD cells

Kristina Engel^1^, Dmitry Chernyakov^1^, Moritz Pernecker^2^ Shobika Karuppusamy^2^, Timm Schreiber^2^ and Bayram Edemir^1,2^

1. Department of Medicine, Hematology and Oncology, Martin Luther University Halle-Wittenberg, Halle (Saale), Germany
2. Department of Physiology and Pathophysiology, Center for Biomedical Education and Research (ZBAF), Witten/Herdecke University, Witten, Germany

* Correspondence to: Bayram Edemir, Department of Medicine, Hematology and Oncology, Martin Luther University Halle-Wittenberg, Ernst-Grube-Str. 40, 06120 Halle (Germany), [bayram.edemir@uk-halle.de](mailto:bayram.edemir@uk-halle.de)

“The authors have declared that no conflict of interest exists.”

**Supplemental Figures**

**Supplementary Figure 1: Clustering of RNA-seq samples based on genotype and tonicity.** (A) Principal component analysis (PCA) of RNA-seq data from mpkCCD cells under isotonic (300 mosmol/kg) and hypertonic (600 mosmol/kg) conditions in scramble control (Scr) and Nfat5 knockout (*Nfat5*-KO) cells. The three principal components explain 23 %, 21 % and 13 % of the total variance, respectively. Each point represents a single biological replicate. (B) Correlation heatmap based on Pearson correlation coefficients. Samples are primarily clustered by osmotic conditions and genotype, confirming the consistency between biological replicates and the strong transcriptomic differences associated with NFAT5 function and tonicity.
